# Appointment of Delegates to the American Medical Association

**Published:** 1863-05

**Authors:** 


### APPOINTMENT OF DELEGATES TO THE AMERICAN MEDICAL ASSOCIATION.

Buffalo Medical Association, Erie County Medical Society, Hospital of the Sisters of Charity, Buffalo General Hospital, the Medical Department of the University of Buffalo, Lying-in Asylum, and Insane Asylum are each entitled to representation in the American Medical Association. We trust the profession here will take action in this matter sufficiently early to insure a full attendance. Many subjects of importance will be presented, in which physicians of the whole country should be deeply interested, and early appointment of suitable delegates should be made.

This National Medical Association has thus far, received the respect and confidence of the profession, and to insure its continuance, the organization itself must be guarded upon all sides from the dangers to which it is exposed. Its action must be above all sectional, political, or personal influences, the interests of the profession—its elevation, protection, and advancement—the only objects of its endeavor. Should it from neglect and inattention, degenerate into a medium of personal advancement or sectional strife, the power of its influence will soon be lost and itself forgotten; while with high purposes and properly conducted efforts, the true interests of the profession will be promoted and confidence and regard continued.

The membership of this Society has been of the most worthy of the profession, and election to office in it regarded as distinguished honor; and whatever action may be taken upon the exciting topics to be presented we hope every attention may be bestowed upon maintaining the integrity of the Society itself. Aspirants for its chief honors are already quite numerous, and we hope that it may be bestowed upon those only who are true to themselves, the profession, and the country; that they may be at least *loya to the country beyond suspicion.* Though we should most heartily oppose all action of a decidedly political bearing, yet we should regard it a disgrace to the profession and to the country, if the chief honors of the Society should be bestowed upon any one *suspected* even, of disloyalty. That such an idea should be suggested will appear strange, that any physician of the North should be suspected disloyal *is very strange.* We have our reasons for calling attention to it.

